# Supplementary figures and images for: Restoring compromised blood-retina-barrier integrity with Netrin-1 overexpression
Source: Cell Mol Life Sci. 2025 Nov 6;82(1):389. doi: 10.1007/s00018-025-05903-6 (PMC12592616; doi:10.1007/s00018-025-05903-6)

# Supplementary Figure 1

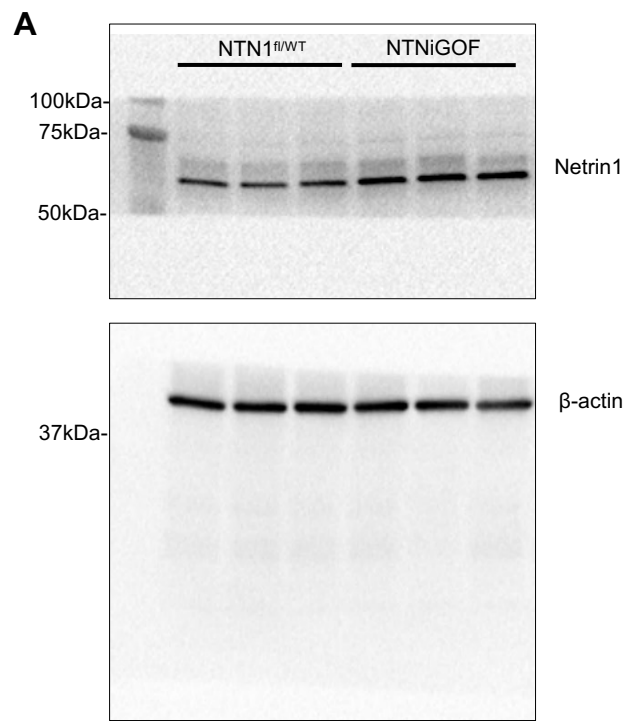

Supplement: Supplementary file 1 — Supplementary file1 (PDF 156 KB) Validation of NTN1iGOF. Uncropped Western blot from Fig.1c shows presence of full-length Netrin-1 and absence of bands below 68kDa [file 18_2025_5903_MOESM1_ESM.pdf]

# Supplementary Figure 2

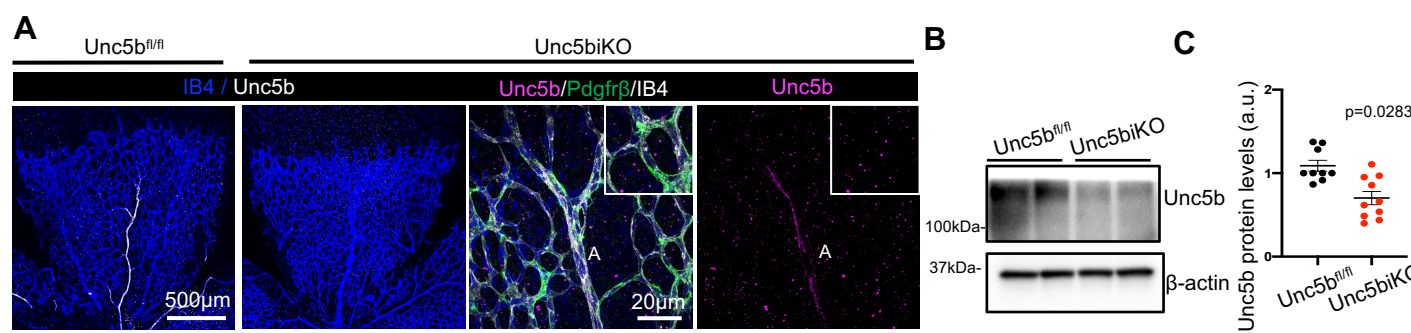

Supplement: Supplementary file 2 — Supplementary file2 (PDF 1160 KB) Validation of global Unc5b deletion. (A) Whole-mount P5 retinas stained with indicated antibodies. (B) Western blot of retina protein extracts and blot quantification (C). Each dot represents one retina from one mouse. Unc5bflfl n=9, Unc5biKO n= 10 [file 18_2025_5903_MOESM2_ESM.pdf]

# Supplementary Figure 3

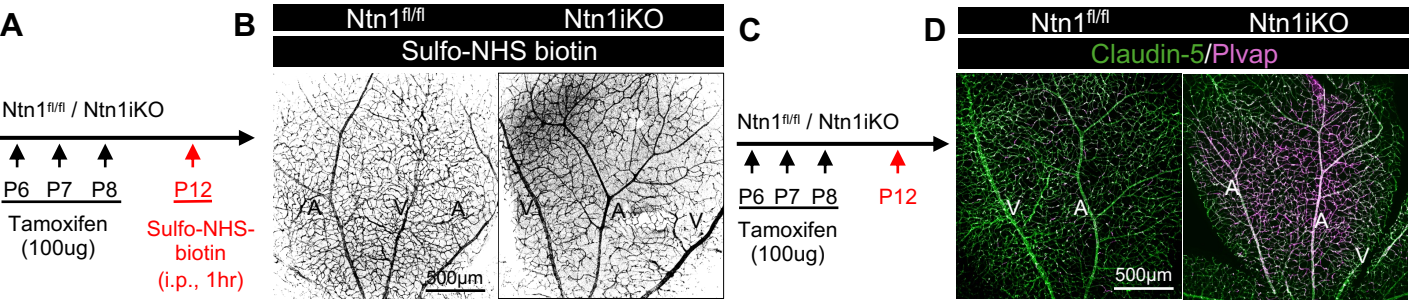

Supplement: Supplementary file 3 — Supplementary file3 (PDF 947 KB) Effects of Ntn1 deletion on BRB integrity. (A) Ntn1 gene deletion and tracer injection strategy. (B). Whole-mount P12 retinas after i.p. injection with sulfo-NHS-biotin for 1h. (C) Ntn1 gene deletion strategy. (D) Whole-mount P12 retinas stained with the indicated antibodies. A: Artery, V: Vein [file 18_2025_5903_MOESM3_ESM.pdf]
